# Nearest Neighbor Networks: clustering expression data based on gene neighborhoods

**DOI:** 10.1186/1471-2105-8-250

**Published:** 2007-07-12

**Authors:** Curtis Huttenhower, Avi I Flamholz, Jessica N Landis, Sauhard Sahi, Chad L Myers, Kellen L Olszewski, Matthew A Hibbs, Nathan O Siemers, Olga G Troyanskaya, Hilary A Coller

**Affiliations:** 1 Department of Computer Science, Princeton University, Princeton, NJ,08544, USA; 2 Lewis-Sigler Institute for Integrative Genomics, Princeton University, Princeton, NJ, 08544, USA; 3 Department of Molecular Biology, Princeton University, Princeton, NJ, 08544, USA; 4 Bristol-Myers Squibb, 31 Pennington-Rocky Hill Road, Pennington, NJ, 08534, USA

## Abstract

**Background:**

The availability of microarrays measuring thousands of genes simultaneously across hundreds of biological conditions represents an opportunity to understand both individual biological pathways and the integrated workings of the cell. However, translating this amount of data into biological insight remains a daunting task. An important initial step in the analysis of microarray data is clustering of genes with similar behavior. A number of classical techniques are commonly used to perform this task, particularly hierarchical and K-means clustering, and many novel approaches have been suggested recently. While these approaches are useful, they are not without drawbacks; these methods can find clusters in purely random data, and even clusters enriched for biological functions can be skewed towards a small number of processes (e.g. ribosomes).

**Results:**

We developed Nearest Neighbor Networks (NNN), a graph-based algorithm to generate clusters of genes with similar expression profiles. This method produces clusters based on overlapping cliques within an interaction network generated from mutual nearest neighborhoods. This focus on nearest neighbors rather than on absolute distance measures allows us to capture clusters with high connectivity even when they are spatially separated, and requiring mutual nearest neighbors allows genes with no sufficiently similar partners to remain unclustered. We compared the clusters generated by NNN with those generated by eight other clustering methods. NNN was particularly successful at generating functionally coherent clusters with high precision, and these clusters generally represented a much broader selection of biological processes than those recovered by other methods.

**Conclusion:**

The Nearest Neighbor Networks algorithm is a valuable clustering method that effectively groups genes that are likely to be functionally related. It is particularly attractive due to its simplicity, its success in the analysis of large datasets, and its ability to span a wide range of biological functions with high precision.

## Background

The availability of DNA microarrays has made it possible to monitor the transcript levels of every mRNA in an entire genome simultaneously. This has allowed researchers to monitor global changes in gene expression that occur in response to a cellular perturbation or the gene expression profiles characteristic of a particular state, such as a tissue type or a disease state. A major goal of integrative genomics is to interpret these gene expression patterns in order to define underlying signaling networks.

As the bulk of publicly available coexpression data has grown, a variety of successful techniques have been proposed for its analysis. In broad terms, these include normalization and meta-analysis [1-4], detection of differential expression [5-7], several forms of clustering [8-11], and many others. However, each time a new microarray data set is produced, it is ultimately in the hands of the generating biologist(s) to inspect the data and to determine what biological insights it might provide. This initial inspection is often aided by classical clustering algorithms such as K-means [12,13] or hierarchical clustering [8,14], both of which are intended to present an intuitive, accessible view of genes whose coexpression might indicate similar regulation or biological functionality.

While these traditional algorithms can serve as a convenient first tool for microarray analysis, they can also be confounded by certain characteristics of biological data. K-means clustering, for example, requires prior knowledge of the number of clusters to find, and it will find that number of clusters even in random data [15]. Similarly, hierarchical clustering is incapable of leaving any genes unclustered, and its results can be driven by strong features in a small number of initially clustered genes [16]. Many more recent clustering algorithms have been proposed to overcome these limitations, with Aerie [17], CAST [18], CLICK [19], GenClust [20], Quality Threshold Clustering (QTC) [9], and SAMBA [21] representing a small cross-section of the tools available for the purpose of coexpression-based gene clustering.

These newer algorithms have overcome the drawbacks of traditional clustering in a number of ways. SAMBA, for example, represents a family of biclustering algorithms capable of excluding conditions as well as genes from a cluster; CLICK and QTC allow genes to remain unclustered, and Aerie and other fuzzy clustering algorithms permit genes to inhabit multiple clusters probabilistically. However, it is unclear how these algorithms perform with respect to their original purpose: providing biologists with a view of coexpressed biological processes within microarray data sets. Given a new data set containing a collection of active biological pathways or functions, do these clustering algorithms accurately group functionally related genes?

We report below a clustering algorithm based on shared nearest neighbors called Nearest Neighbor Networks (NNN) intended to serve as a useful tool for biologists when discovering functional activity in coexpression data sets. Although NNN shares some features (such as the identification of subgraphs with high connectivity) with existing graph-theoretic clustering techniques [22,23], it is unique in its focus on groups of genes sharing a mutual nearest neighborhood (based on some distance or similarity measure) rather than on groups of genes that are tightly correlated in some absolute measure, and NNN goes beyond simple clique finding to produce complex, biologically relevant clusters. We present the results of a functional evaluation [24] demonstrating NNN's ability to retrieve precise clusters that represent the diverse biological activity present in six qualitatively different microarray data sets. This evaluation also examines the behavior of the eight clustering algorithms discussed above to determine their accuracy in producing related gene clusters from many types of coexpression data and within many biological processes. Additionally, we compare the behavior of these clustering algorithms when presented with random data and when extracting clusters from integrated data (i.e. from a merged collection of all six microarray data sets). We believe that NNN represents an intuitive, simple tool providing biologists with a way to rapidly obtain and visualize a comprehensive collection of the processes coexpressed in a microarray data set.

## Implementation

### NNN algorithm

In designing a clustering algorithm that would allow us to make highly coherent clusters, we were inspired by the approach taken by Stuart and colleagues to define the homologues of a specific gene in multiple species [25]. In Stuart et al, a metagene was defined as a set of genes across multiple organisms whose protein sequences are one another's best reciprocal BLAST hits. These metagenes were then grouped into an interaction network (without being clustered) using an aggregate similarity score measuring correlation under many diverse microarray conditions. In contrast, the NNN clustering algorithm begins with an interaction network defined by a standard similarity measure (such as Pearson correlation or Euclidean distance between two genes' expression vectors) and finds clusters by extracting small cliques of mutual nearest neighbors (akin to best reciprocal hits). We then group together cliques that overlap to form larger clusters of genes.

NNN receives as input a set of genes of size *m*, a similarity measure *d*(*g*_1_, *g*_2_), a clique size *g*, and a neighborhood size *n*. Its output is an assignment of each gene to zero or more clusters.

**Figure 1:**
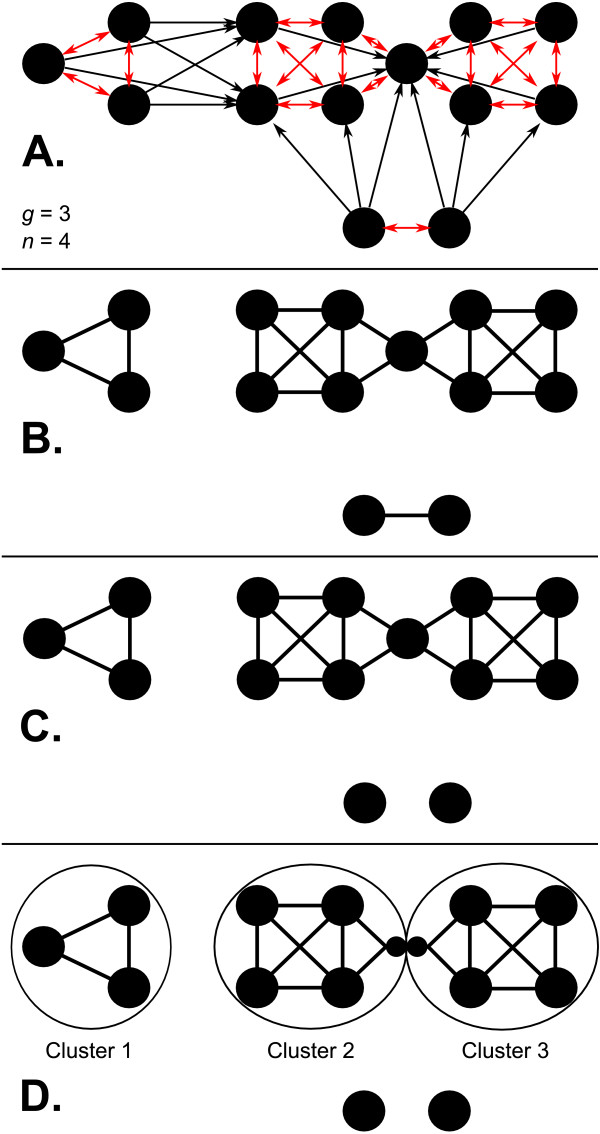
**NNN Algorithm overview**. An example of the Nearest Neighbor Networks operating on 14 genes with clique size *g *= 3 and neighborhood size *n *= 4. A. A directed graph is generated in which each gene is connected to its *n *nearest neighbors. B. An undirected graph is constructed from bidirectional connections. C. Overlapping cliques of size *g *are merged to produce preliminary networks. D. Preliminary networks containing cut-vertices are split into final networks, with copies of the cut-vertices occupying both networks.

For each gene *g*_*i*_, the *n *nearest neighbors *N*(*g*_*i*_) = {*g*_*i*,1_, ..., *g*_*i*, *n*_} are calculated based on the similarity measure *d*. If genes are considered to be vertices in a graph, this results in a directed graph in which each node is of out degree *n *(Figure 1A). An undirected graph is then constructed by connecting any two genes *g*_*i *_and *g*_*j *_such that *g*_*i *_∈ *N*(*g*_*j*_) and *g*_*j *_∈ *N*(*g*_*i*_), i.e. the two genes are mutual nearest neighbors (Figure 1B). All cliques (complete subgraphs) of size *g *within this graph are identified, and overlapping cliques are merged to produce preliminary networks representing potential clusters of related genes (Figure 1C).

A small number of genes in any genome often serve as interaction hubs connecting a large collection of minimally related partners [26], and these genes can cause NNN to merge cliques to an undesirable extent. To address this issue, NNN uses a well-established algorithm to remove cut-vertices in its preliminary networks [27-29]. A cut-vertex is a node whose removal results in an additional disconnected component in a graph; in our preliminary networks, such nodes represent genes connecting clusters which share no other interactions and are thus likely to be functionally irrelevant interaction hubs. Each of our preliminary networks is divided at its cut-vertices into multiple final networks, and the cut-vertices are included in each of the two networks which they induce (Figure 1D). Finally, to further ensure that cliques are not merged undesirably, any network (at most one) containing more than half of the input genes is removed.

NNN runtimes are generally below five minutes with reasonable parameter settings on a modern computer; with *g *= 5 and *n *= 25, the Hughes data set (the largest used in our analysis) is fully clustered in approximately three minutes running in a single thread on a 2 GHz Core 2 Duo processor. Clustering with a worst-case *g *= 5 and *n *= 40 takes approximately 11.5 minutes, and the lower bound *g *= 3 and *n *= 10 runs in under 2.5 minutes. In the latter case, most of this time is spent calculating gene pair correlations. See Supplementary Figure 1 for more information on NNN runtimes.

### Microarray data processing

To evaluate the abilities of NNN and other clustering algorithms to accurately cluster functionally related genes across a range of biological processes, we ran them on six *Saccharomyces cerevisiae *microarray data sets [30-35]. The data sets range from seven to 300 conditions, include Agilent, Affymetrix, and custom cDNA arrays, include both time course and isolated measurements, and span a wide variety of biological perturbations and conditions.

In all cases save Haugen et al (who provide data that has already been preprocessed), the data sets were filtered to remove genes with more than 50% missing data. Any remaining missing values were imputed using KNNImpute [36] with *k *= 10, and replicated genes were averaged to ensure that each data set contained at most one expression vector per open reading frame. For single channel data, expression values less than two were considered to be missing, and all single channel values were logarithmically transformed as a final preprocessing step. The two replicates in Brem et al were averaged together.

In order to construct a merged data set consisting of conditions from all six individual microarray data sets, a data matrix was constructed containing each gene present in any of the data sets. Genes were assigned missing values for data sets in which they were not present. This merged data matrix was filtered to remove genes missing data for 50% or more of the resulting 664 conditions, and any remaining missing values were imputed using KNNImpute with *k *= 10. This left 6160 genes, each represented by an expression vector of length 664 containing no missing values.

### Random data generation

Randomized synthetic data was generated to characterize the behavior of NNN and other clustering algorithms when presented with data containing clusters present only by chance. Two sets of randomized data were generated, both containing 6000 "genes" and 10 conditions. In the uniform case, each data value was drawn uniformly from the range [-1, 1]. In the normally distributed data sets, each value was drawn from *N*(0, 1). Five data sets of each type were generated and used for the evaluations discussed below.

### Evaluation methods

In order to determine the accuracy and coverage of the functional relationships predicted by these clustering methods, we employed an evaluation method similar to that described in [37]. Specifically, we used the same 200 functions drawn from the Gene Ontology [38] as sets of "known" related genes; genes coannotated below these terms were considered to be functionally related. To generate negative examples, any gene pairs not coannotated below some GO term including at least 10% of the *S. cerevisiae *genome (roughly 645 genes) were considered to be unrelated. This resulted in an answer set of 620854 related and 8531975 unrelated pairs.

Each clustering method was evaluated by considering any gene pair sharing a cluster to be related and any gene pair clustered separately to be unrelated; unclustered genes (when applicable) were neither related nor unrelated. This process transforms any clustering result into a set of related and unrelated gene pairs from which we calculated precision, recall, and/or area under an ROC curve (AUC) relative to the answer set. When performing per-biological function evaluations, these measures were calculated over subsets of the global answer set relevant to each function of interest; specifically, a gene pair was considered relevant to some function if i) it represented a positive relationship and both genes were included in the function or ii) it represented a negative relationship and one gene was included in the function [24]. All AUCs were calculated analytically using the Wilcoxon Rank Sum formula [39].

### Evaluation parameters

Where possible, we evaluated each clustering algorithm over a range of parameters, e.g. K-means for values of *k *ranging from two to 30. By recording the most restrictive parameter setting at which any gene pair clustered together, we were able to generate full precision/recall curves for most clustering methods. In cases where this was not possible, a single clustering was generated per data set, resulting in a point rather than a curve (but not affecting AUC calculations). All applicable clustering algorithms used Pearson correlation as a similarity measure.

Nearest Neighbor Networks was evaluated using our own Java implementation with the neighborhood size parameter *n *ranging from one to 30 in increments of three. The maximum neighborhood size used with the concatenated data set for the per-function evaluation was increased to 40 in order to provide coverage of a greater number of Gene Ontology terms. In all functional evaluations, the clique size *g *was fixed at five. The effects of varying *g *can be seen in Supplementary Figure 2, with larger values slightly increasing precision while becoming more computationally expensive ([40], Supplementary Figure 1).

The K-means, CLICK, and SAMBA algorithms were evaluated using the implementation provided by the Expander tool [19]. For K-means, *k *was varied from two to 30 by increments of two. The CLICK and SAMBA algorithms were run with the default parameters provided by Expander, resulting in a single clustering. The predicted cluster confidences produced by SAMBA were used in lieu of a parameter setting to determine cluster specificity, with a higher confidence indicating a more specific cluster.

TIGR MeV [41] was used to execute the CAST algorithm, with the threshold parameter varied from 0.5 to 0.9 by increments of 0.05. Our own C++ implementation of Quality Threshold Clustering was used with a minimum cluster size of five and diameters ranging from 0.05 to 0.8 by increments of 0.05. QTC was unable to evaluate the concatenated data set due to its reliance on the computationally intensive jackknife distance measure [9]. Our own implementation of Pearson correlation was used as a representation of hierarchical clustering, with the raw pairwise correlation value itself behaving as a parameter over which precision and recall were calculated.

Implementations of GenClust and Aerie were provided by [20] and [17], respectively. GenClust was run for 1000 iterations with cluster counts *k *ranging from two to 30 by increments of two. GenClust failed to produce any output for the Hughes or concatenated data sets, apparently due to their high condition counts. Aerie was executed with *k *ranging from 10 to 40 by increments of two, as it failed to produce results for any *k *below 10. Aerie would not operate on the Primig data set regardless of parameter settings, and produced output for the Haugen data set only for *k *up to 22. Since Aerie's *k *does not correspond to a final cluster count, each gene was assigned a vector of centroid distances corresponding to different initial *k*s, and gene pair similarities were calculated as correlations between these vectors.

## Results and discussion

As shown below, NNN succeeds in producing small, precise clusters from coexpression data, and these clusters generally span a wider variety of biological processes than those produced by the other clustering algorithms evaluated. While NNN's recall is lower than that of clustering algorithms in which all genes are always clustered, the capability to leave genes unclustered allows NNN to present an analyst with results consisting of only the high precision results of biological interest. This is evidenced, for example, in NNN's behavior when run on random data, which is often left unclustered (Table 1, Supplementary Table 1). Furthermore, as described below, NNN produces clusters with substantial functional diversity; particularly on larger data sets, NNN detects activity in processes such as *conjugation *and *phosphorus metabolism *not captured by other clustering algorithms.

**Table 1:** Clustering algorithm summary statistics.

	**NNN ***g *= 5, *n *= 25	**CAST ***t *= 0.8	**CLICK ***h *= μ_T_	**QTC ***d *= 0.5, *n *= 5	**SAMBA**
*Brem 2005, 6162 genes, 131 conditions*					

**Genes**	1527	3410	6162	6137	2284
**Clusters**	54	800	82	127	113
**Mean Size**	28.4	4.26	75.1	48.3	102
**Size Dev.**	49.2	16.91	161	93.3	70.3

*Gasch 2000, 6115 genes, 173 conditions*					

**Genes**	1142	4079	6115	6092	3120
**Clusters**	38	666	9	69	128
**Mean Size**	30.1	6.12	679	88.3	130
**Size Dev.**	62.5	35.58	787	220	101

*Haugen 2004, 6256 genes, 7 conditions*					

**Genes**	64	6251	6256	6236	280
**Clusters**	11	45	16	56	5
**Mean Size**	5.82	138.9	391	11.4	88.4
**Size Dev.**	1.19	347.3	474	258	36.5

*Hughes 2000, 6153 genes, 300 conditions*					

**Genes**	1996	2579	6153	6121	3375
**Clusters**	29	519	75	177	325
**Mean Size**	68.9	4.97	82.0	34.6	45.9
**Size Dev.**	245.4	11.95	107	57.8	44.1

*Primig 2000, 6005 genes, 24 conditions*					

**Genes**	2247	5820	6005	5970	778
**Clusters**	27	687	46	110	25
**Mean Size**	83.2	8.47	131	54.3	139
**Size Dev.**	390	19.26	187	80.4	96.3

*Spellman 1998, 5701 genes, 25 conditions*					

**Genes**	2050	5535	5701	5669	777
**Clusters**	28	616	47	100	32
**Mean Size**	73.3	8.99	121	56.7	69.0
**Size Dev.**	324	30.14	206	114	37.3

*Concatenated Data, 6160 genes, 660 conditions*					

**Genes**	694	6155	6160	-	4892
**Clusters**	29	7	5	-	609
**Mean Size**	23.9	879.3	1232	-	63.7
**Size Dev.**	34.7	2140	1768	-	82.0

*Uniformly Distributed Random Data, 6000 genes, 10 conditions*					

**Genes**	0 (± 0)	5988 (± 0.89)	3600 (± 3286)	5964 (± 28.8)	0 (± 0)
**Clusters**	0 (± 0)	216.2 (± 2.95)	9.8 (± 9.81)	109 (± 4.72)	0 (± 0)
**Mean Size**	0 (± 0)	27.7 (± 0.38)	190 (± 175)	53.0 (± 1.39)	0 (± 0)
**Size Dev.**	0 (± 0)	21.86 (± 0.25)	48.8 (± 45.7)	35.2 (± 0.791)	0 (± 0)

*Normally Distributed Random Data, 6000 genes, 10 conditions*					

**Genes**	0 (± 0)	5986 (± 3.58)	6000 (± 0)	5975 (± 4.77)	0 (± 0)
**Clusters**	0 (± 0)	231.6 (± 3.29)	28.8 (± 11.9)	124 (± 1.30)	0 (± 0)
**Mean Size**	0 (± 0)	25.85 (± 0.36)	235 (± 82.6)	48.3 (± 0.482)	0 (± 0)
**Size Dev.**	0 (± 0)	18.14 (± 0.15)	64.8 (± 46.3)	30.9 (± 0.374)	0 (± 0)

*Brem 2005, 6162 genes, 131 conditions, randomly permuted*					

**Genes**	101.4 (± 28.85)	0 (± 0)	6162 (± 0)	5837 (± 260.6)	1061 (± 35.87)
**Clusters**	16.2 (± 3.96)	0 (± 0)	36.2 (± 28.99)	428 (± 33.88)	156 (± 4.85)
**Mean Size**	6.23 (± 0.78)	0 (± 0)	680.7 (± 864.7)	13.67 (± 0.46)	32.46 (± 1.35)
**Size Dev.**	1.64 (± 0.79)	0 (± 0)	884.5 (± 1179)	2.36 (± 0.52)	18.03 (± 1.13)

*Gasch 2000, 6115 genes, 173 conditions, randomly permuted*					

**Genes**	19.4 (± 6.66)	0 (± 0)	4586 (± 3058)	5507 (± 47.19)	1382 (± 15.27)
**Clusters**	3.6 (± 1.34)	0 (± 0)	20.75 (± 33.71)	411.2 (± 5.12)	219.8 (± 15.27)
**Mean Size**	5.47 (± 1.04)	0 (± 0)	701 (± 941.3)	13.39 (± 0.058)	18.38 (± 0.35)
**Size Dev.**	0.66 (± 1.48)	0 (± 0)	950.8 (± 1197)	1.7 (± 0.03)	9.25 (± 0.38)

*Hughes 2000, 6153 genes, 300 conditions, randomly permuted*					

**Genes**	20.2 (± 8.61)	572.8 (± 12.74)	4922 (± 2752)	4815 (± 76.96)	1808 (± 56.32)
**Clusters**	3.6 (± 1.82)	224 (± 8.22)	13 (± 10.84)	407.2 (± 7.56)	390.8 (± 5.67)
**Mean Size**	6.13 (± 1.64)	2.56 (± 0.044)	592.5 (± 826.8)	11.83 (± 0.038)	11.09 (± 0.39)
**Size Dev.**	0.53 (± 0.71)	0.82 (± 0.046)	101.7 (± 200.2)	1.15 (± 0.024)	5.59 (± 0.5)

Summary statistics detailing Nearest Neighbor Networks clusters formed from the data sets employed in this study, from their concatenation, and from two synthetic random data sets using default parameters (*g *= 5, *n *= 25). Results from other clustering algorithms with appropriate output formats (CAST, CLICK, QTC, and SAMBA) have been included, also utilizing default parameter settings provided by the algorithms' implementations. Random values are shown with standard deviations over five different seeds.

### Nearest Neighbor Networks

**Figure 2:**
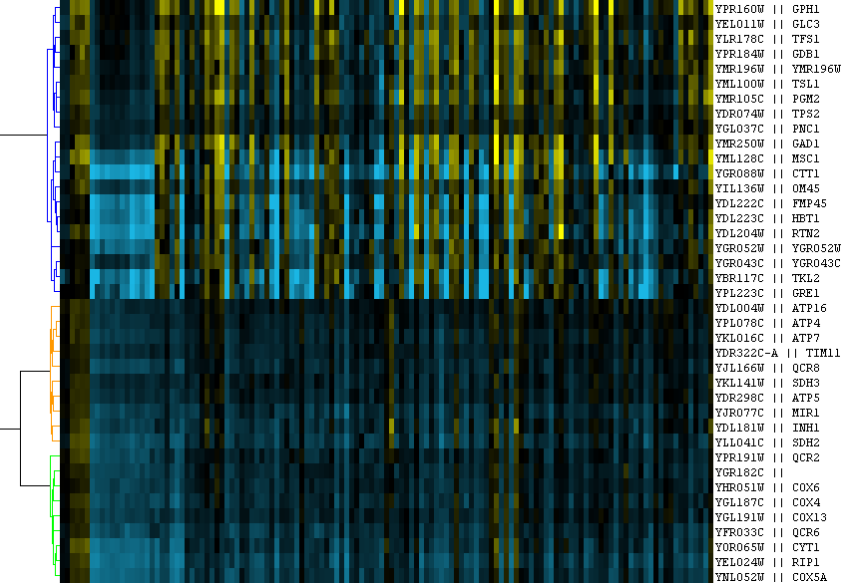
**Example NNN output**. A subset of the Nearest Neighbor Networks clusters produced from the [35] data set using the parameters *g *= 5 and *n *= 10, visualized using Java TreeView [42]. NNN clusters have been colored, internally hierarchically clustered, and the cluster centroids have in turn been hierarchically clustered to provide an easily interpretable tree.

NNN is intended to be an accessible and convenient tool for rapidly producing functionally coherent clusters from coexpression data, and visualization is therefore an important aspect of its results. Figure 2 demonstrates a sample of the default NNN output format as visualized by Java TreeView [42]. Here, each colored subtree represents a cluster found by NNN; these have been internally hierarchically clustered using standard correlation and average linkage for visual coherence, and the clusters centroids have in turn been clustered to produce a full tree. Our NNN implementation also provides a tabular output format assigning genes to numbered clusters for further computational processing.

### Global evaluation of clustering algorithms

**Figure 3:**
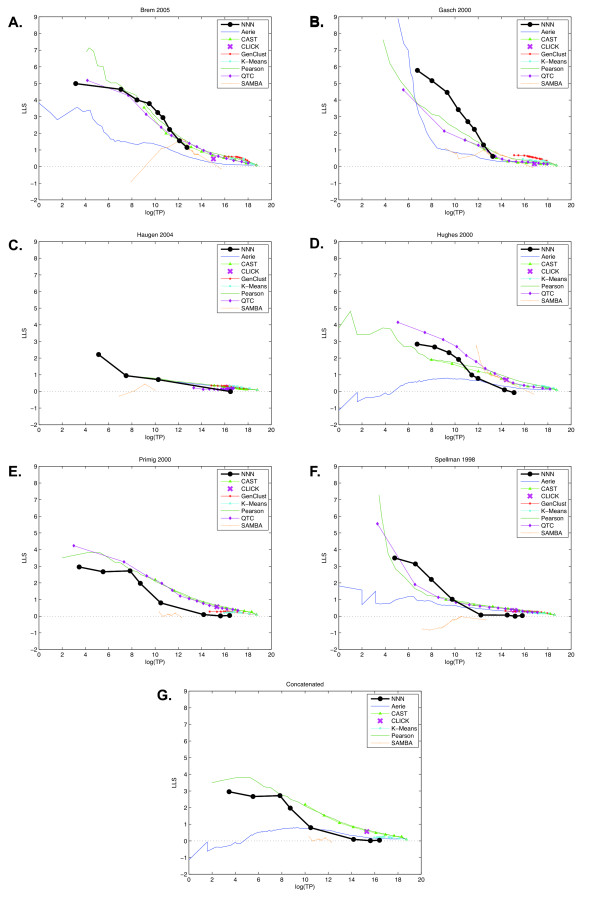
**Global evaluation of clustering algorithms**. Evaluation results for eight clustering algorithms and six microarray data sets based on the global answer set (employing 200 GO terms of functional interest and discarding *ribosome biogenesis and assembly *[37]). Performance has been measured using log_2_(*TP*) on the horizontal axis and log-likelihood score *LLS *= log_2_((*TP*/*FP*)/(*P/N*)) for *P *total positive pairs, *N *total negative pairs, and *TP *and *FP *the number of true and false positives at a particular recall threshold. A. Brem 2005. B. Gasch 2000. C. Haugen 2004. D. Hughes 2000. E. Primig 2000. F. Spellman 1998. G. All six data sets concatenated.

A global evaluation of NNN and eight other clustering algorithms (employing a wide range of parameter settings) on each of the six microarray data sets appears in Figure 3. As recommended in [37], we have excluded the Gene Ontology term *ribosome biogenesis and assembly *during these evaluations so as not to bias the outcome towards this function. Myers et al discusses the problems raised in coexpression analysis by ribosomal genes, in particular their tendency to correlate so strongly even across conditions unrelated to ribosomal functions that they can obscure other biological activity. Especially in data sets eliciting strong stress responses (e.g. Figure 3B), this has a substantial impact on many of the clustering methods, accounting for a portion of their low performance and indicating that they may be clustering more easily discovered ribosomal genes at the expense of genes coexpressed for other biological reasons. Similarly, Supplementary Figure 3 details performance when gene pairs with high sequence similarity are also removed, which has a negligible impact on the evaluation.

Although no one clustering algorithm is appropriate for every situation, Nearest Neighbor Networks demonstrates a clear advantage in precision in many of these data sets. In particular, the Gasch, Haugen, and Spellman data sets are perhaps best analyzed by NNN, demonstrating a robustness to functional bias [37], low condition count, and periodicity, respectively. NNN performs approximately equivalently to QTC and Pearson correlation on the Brem data set, and the Aerie, CAST, and SAMBA algorithms fall slightly beneath these due mainly to precision issues at low recall. CLICK is difficult to evaluate in this context due to its insensitivity to homogeneity parameter changes, leaving no way to trade off between precision and sensitivity. Thus, in a variety of contexts, NNN is best able to extract functionally relevant clusters from coexpression data with high precision.

NNN falls slightly short of QTC and, to a lesser extent, Pearson correlation in the Primig data set, and QTC and SAMBA are both strong performers on the Hughes data. This latter effect might be attributable to the unordered nature of the Hughes data (a deletion study rather than a time course) from which SAMBA is able to bicluster correlated conditions as well as genes, and the large condition count likely benefits both SAMBA and QTC. NNN's performance in the high precision/low recall region of the Primig data set is impaired by the fact that the Gene Ontology annotates MATALPHA1 and HMLALPHA under the *development *term, STE14 under the *protein processing *term, and STE3 and MF(ALPHA)1 under the *reproduction *term. This results in our answer set considering their pairwise combinations (e.g. MATALPHA1 with STE14, STE14 with STE3, and so forth) to be unrelated, while NNN predicts them to be tightly clustered together.

While NNN is never more than slightly below the best performing algorithms, certain specific issues with other methods become apparent from this type of functional analysis. For example, SAMBA has some difficulty with the extremely small Haugen data set (Figure 3C) and the periodic Spellman cell cycle data (Figure 3F).

Table 1 provides summary statistics describing the output of NNN using default parameters of *g *= 5 and *n *= 25 on the six data sets evaluated more fully below, on the concatenation of those six data sets, and on random synthetic and permuted data. For purposes of comparison, similar statistics have been provided from other clustering algorithms (where applicable) using their default parameter settings. NNN, QTC, and SAMBA are capable of leaving genes unclustered; CAST does not explicitly leave genes unclustered, but it does generate clusters of size one, effectively removing any such gene from the clustering. NNN and SAMBA succeed in taking advantage of this trait to recognize and ignore synthetic random data. Both NNN and SAMBA also deal well with randomly permuted data, the former responding particularly well (i.e. leaving most genes unclustered) to data sets with many conditions and the latter to data sets with few. CAST responds to large randomized data sets extremely well, and while it still clusters many genes in random or randomized data, it does generate characteristically small clusters that an analyst could likely detect. With default parameter settings, NNN tends to be conservative, generally producing fewer, smaller, and (as evaluated above) more precise clusters than CAST or SAMBA.

Note that the default parameters may not be appropriate for all analyses; they are used here for comparison purposes. For example, more clusters can be obtained from the Haugen or concatenated data sets (if desired) by increasing *n*. The global evaluation above and functional evaluations below cover a wide range of parameter settings for all clustering methods and show results largely independent of specific parameter values.

### Behavior on random data

It is of interest to note that only Nearest Neighbor Networks, SAMBA, and, in one case, CAST succeed in excluding randomized data from their clustering output. SAMBA achieves this by computing the statistical significance of bicluster weights and retaining only those unlikely to occur by chance [43]. NNN instead takes advantage of the fact that random data of this form tends to over-cluster, i.e. for an appropriate neighborhood size, all or nearly all genes cluster together. Since substantially overlarge clusters are eliminated by NNN, this results in the removal of randomized data from the functional clusters provided to the user.

### Behavior on concatenated data

Only NNN and Pearson correlation succeed in extracting functional relationships from the concatenated data sets, with NNN achieving somewhat better recall. As discussed in [24], algorithms relying solely on correlation measured over a long expression vector can be easily misled. This can be caused by differences in normalization between the data sets making up the concatenated vector or by overriding "global" signals providing high correlation among only a small set of ubiquitously coexpressed genes (e.g. the ribosomal genes discussed above). This has the effect of producing a small number of very highly correlated genes and relegating most of the correlations of functional interest to near-background levels. NNN avoids this problem by regarding both tight and diffuse clusters as equally valid, so long as cliques of mutual nearest neighbors are present.

For example, consider a group of ribosomal proteins coexpressed across all conditions with a mutual correlation of 0.9. A group of meiotic genes only activated under specific circumstances might achieve a correlation of 0.3 when tested across many conditions, since they will not usually be coregulated. If functionally unrelated genes tend to correlate at a level of 0.2, the ribosomal cluster will be far easier to discover. However, NNN will not distinguish between absolute correlation levels so long as the genes in each group are within each others' nearest neighborhoods – which will likely be the case, since their mutual correlations remain above background. Meta-analytic normalization techniques provide another solution to this problem; correlations combined by z-scoring substantially outperform raw correlations, and these z-scores are in turn outperformed by NNN clustering using z-scores in place of Pearson correlation as input (data not shown).

### Functional evaluation of clustering algorithms

**Figure 4:**
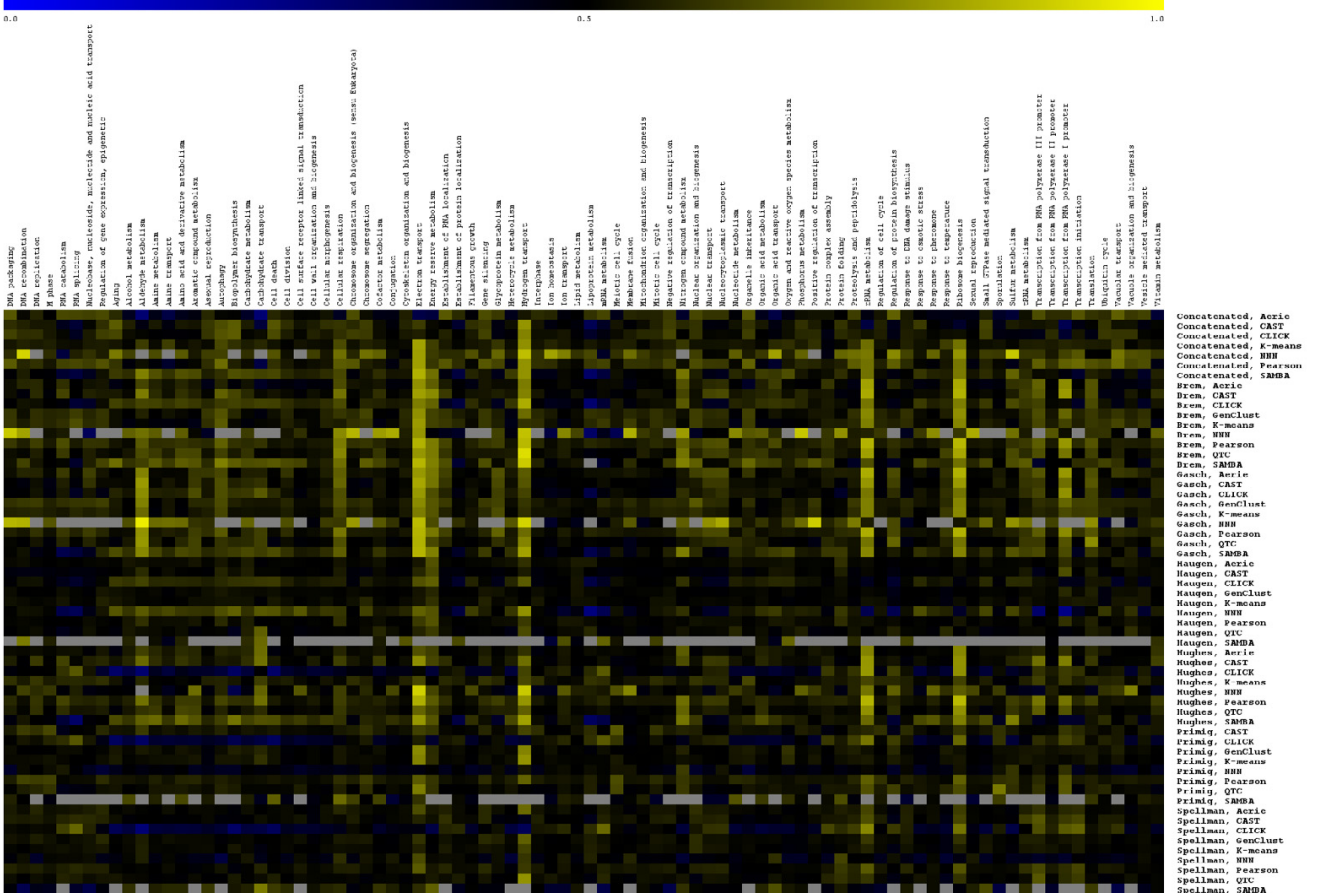
**Functional evaluation of clustering algorithms**. Function-specific evaluation results for each clustering method on a per data set and GO term basis. Each cell represents an AUC score calculated analytically using the Wilcoxon Rank Sum formula; below baseline performance appears in blue, and yellow indicates higher performance. Data set and term combinations for which ten or fewer pairs were able to be evaluated are excluded and appear as gray missing values; functions for which less than 10% of methods were available due to gene exclusion by NNN, QTC, or SAMBA were removed. Visualization provided by TIGR MeV [41].

A global evaluation such as the one described above does not reveal the functional diversity of the predicted interactions; even with ribosomal interactions removed, it is possible for an algorithm to perform well by accurately predicting only a few biological processes. A complementary functional evaluation demonstrates that Nearest Neighbor Networks not only performs approximately as well or better than other clustering methods in global evaluations, it produces clusters which capture a wider array of biological functions. The heat map in Figure 4 indicates AUC scores for a variety of Gene Ontology terms within each data set. NNN succeeds in accurately predicting clusters for several terms poorly analyzed by other algorithms, particularly within the Brem and Gasch data sets (Supplementary Figure 4).

The high predictive power of Nearest Neighbor Networks in the Brem data set likely reflects the unique nature of these microarray conditions. This data set includes gene expression profiles from the segregants of a cross between two different strains of yeast. As opposed to most data sets, in which haploid yeast of one mating type are profiled, segregants with both the MATA and the MATALPHA phenotypes were present in the Brem data, making it possible to identify other genes correlated with mating type. In addition, there is a polymorphism between the parental strains in the pheromone response G protein GPA1, which is expected to result in differences in expression of effector genes among the segregants. Further, an interaction between the mating-type locus MAT and the pheromone response gene GPA1 has been detected [35]. The expression profiles of genes in the *response to pheromone*, *sexual reproduction*, and *conjugation *functions are consequently related in this data set and provide an opportunity for identifying high precision networks of genes with these Gene Ontology annotations.

**Figure 5:**
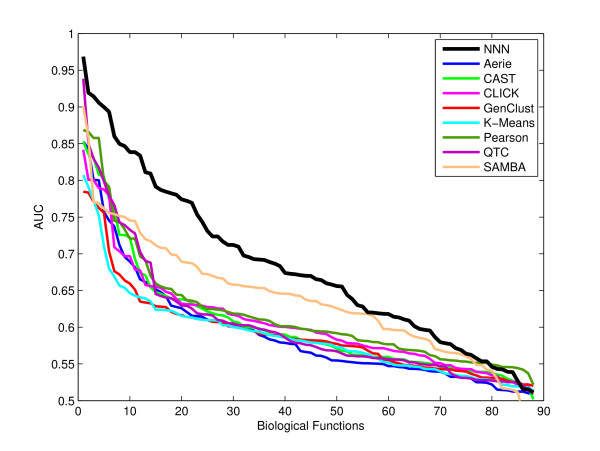
**Functional diversity of clustering algorithms**. An evaluation of each clustering algorithm's ability to detect the 88 biological processes for which data was available in our analysis. For each algorithm, the maximum AUC across all six data sets was determined, and the resulting AUCs are presented here in descending order per algorithm. NNN correctly clusters genes from substantially more biological processes relative to previous methods.

NNN clusters tend to describe a broader array of biological processes than those of previous methods, and they often relate functional information that might otherwise remain undetected. Figure 5 summarizes each clustering algorithm's maximum performance for each biological function across all six data sets. Of the 88 functions evaluated in this manner, 40 are predicted at biologically uninformative levels (AUC < 0.65) by previous methods. NNN improves 18 of these functions to an AUC greater than 0.65 (as high as 0.9 in several cases). It further improves performance in an additional 21 functions also predicted well (AUC > 0.65) by other algorithms. In the concatenated data, NNN improved the best AUC above 0.65 in 14 functions and was the best predictor of an additional 10 beyond those. As Figure 5 indicates, NNN is generally able to recover information about more biological processes with higher precision than other clustering algorithms.

By considering the detailed functional breakdown in Figure 4, it is possible to comment on some qualitative aspects of this improvement. For example, there are several small groups of related GO terms for which NNN provides a consistent improvement across two or more data sets. These include the mating response (*membrane fusion*, *conjugation*, *sexual reproduction*, and *response to pheromone*), metabolism of various nutrients (*phosphorus*, *sulfur*, and *alcohol metabolism*) and cellular building blocks (*amine*, *amino acid*, *organic acid*, and *nucleotide metabolism*), and cellular respiration (*electron *and *hydrogen transport *and *cellular respiration*), among others. A unifying theme among these processes is that they are all carried out by relatively small sets of genes coexpressed only under specific conditions. Only a small number of the Brem segregants or Hughes deletion mutants, for example, might disrupt the 60 genes annotated to *sulfur metabolism*, and despite being coordinately disrupted, this will only change the affected genes' pairwise similarities by a small amount. This small change is difficult to detect by clustering algorithms that consider absolute similarities, but because these genes have all moved mutually closer together, NNN is more likely to place them in each others' nearest neighborhoods despite the "diffuse" quality of those neighborhoods. Particularly since specific functions such as these can represent the most biologically interesting effects of a microarray experiment, it is critical to provide a method such as NNN which will extract the most precise and functionally diverse clusters from a data set.

## Conclusion

We present the Nearest Neighbor Networks clustering algorithm as an efficient and convenient tool for extracting precise, functionally diverse clusters from coexpression data. NNN leaves less active genes unclustered and focuses on networks of potential interaction rather than on minimizing distances; this results in smaller clusters with a high degree of functional relationship as measured by known annotations in the Gene Ontology. Particularly in complex data sets for organisms without comprehensive reference data readily available, NNN's more precise clusters should be beneficial in coexpression analysis (see Supplementary Figure 5 for a sample clustering of human data). Moreover, these clusters span a wider range of biological processes than those typically extracted from microarray data sets by other clustering algorithms. We hope that these features will allow NNN to serve as a useful method for biologists to obtain an overview of the genes and processes active in new data sets.

## Availability and requirements

Project name: Nearest Neighbor Networks

Project home page: 

Operating System: Platform independent

Programming language: Java

Requirements: Java 1.5 or greater

License: Creative Commons Attribution 2.5

## Authors' contributions

CH, AIF, JNL, NOS, and HAC contributed to the algorithm; CH, AIF, and SS contributed to the implementation; CH, JNL, CLM, KLO, MAH, OGT and HAC contributed to the analysis of the clustering output; CH and HAC wrote the paper with input from the other authors. All authors approved the final version.
